# Characterization of the novel mitochondrial genome replication factor MiRF172 in *Trypanosoma brucei*

**DOI:** 10.1242/jcs.211730

**Published:** 2018-04-25

**Authors:** Simona Amodeo, Martin Jakob, Torsten Ochsenreiter

**Affiliations:** 1 Institute of Cell Biology, University of Bern, Bern, Switzerland; 2 Graduate School for Cellular and Biomedical Sciences, University of Bern, Bern CH-3012, Switzerland

**Keywords:** Maxicircles, Minicircles, Minicircle replication factor 172, Mitochondrial DNA replication, Tripartite attachment complex, TAC, *Trypanosoma brucei*

## Abstract

The unicellular parasite *Trypanosoma brucei* harbors one mitochondrial organelle with a singular genome called the kinetoplast DNA (kDNA). The kDNA consists of a network of concatenated minicircles and a few maxicircles that form the kDNA disc. More than 30 proteins involved in kDNA replication have been described. However, several mechanistic questions are only poorly understood. Here, we describe and characterize minicircle replication factor 172 (MiRF172), a novel mitochondrial genome replication factor that is essential for cell growth and kDNA maintenance. By performing super-resolution microscopy, we show that MiRF172 is localized to the kDNA disc, facing the region between the genome and the mitochondrial membranes. We demonstrate that depletion of MiRF172 leads to a loss of minicircles and maxicircles. Detailed analysis suggests that MiRF172 is involved in the reattachment of replicated minicircles to the kDNA disc. Furthermore, we provide evidence that the localization of the replication factor MiRF172 not only depends on the kDNA itself, but also on the mitochondrial genome segregation machinery, suggesting an interaction between the two essential entities.

This article has an associated First Person interview with the first author of the paper.

## Abstract (precis)

**Highlighted Article:** MiRF172 is a novel protein involved in the reattachment of replicated minicircles in *Trypanosoma brucei*; this protein requires the mitochondrial segregation machinery for correct localization.

## INTRODUCTION

We find one of the most intriguing genome organizations in the mitochondrial genome of Kinetoplastea, which is a class of single-celled eukaryotes. The name Kinetoplastea refers to the single mitochondrial genome (kinetoplast DNA, kDNA) of the organism, which, in most cases, is positioned close the base of the flagellum. Even by 1913 Muriel Robertson speculated on whether the blepharoplast (now called the basal body) and the kinetonucleus (now kinetoplast) in trypanosomes (Robertson, 1913). Later, several laboratories demonstrated the physical connection of the flagellum to the kDNA in *Leishmania* and *Trypanosoma* using biochemical and cell biological approaches (Laurent and Steinert, 1970; Robinson and Gull, 1991; Simpson, 1968). In 2003, an electron microscopy study visualized the underlying structure in *T. brucei* and it was named the tripartite attachment complex (TAC) (Ogbadoyi, 2003). Several proteins belonging to this structure have now been identified that indicate that the organization of the segregation machinery is complex (Gheiratmand et al., 2013; Käser et al., 2016, 2017; Schnarwiler et al., 2014; Trikin et al., 2016; Zhao et al., 2008).

The mitochondrial genome itself is composed of small and larger plasmid like elements referred to as the mini- and maxicircles, respectively. Maxicircles [23 kilobase (kb) in *T. brucei*] encode 18 protein-coding and two ribosomal RNA genes and are the functional homologs of other mitochondrial genomes. The majority of the mitochondrial genes are cryptic and require post-transcriptional editing to code for the bona fide components of the respiratory chain and a ribosomal protein (Hajduk and Ochsenreiter, 2010; Jensen and Englund, 2012; Povelones, 2014). The editing process is mediated by large protein complexes (Aphasizheva and Aphasizhev, 2016; Göringer, 2012; McDermott et al., 2016) and small non-coding guide RNAs (gRNAs) (Blum et al., 1990). These gRNAs are transcribed from the minicircles, which in *T. brucei* are 1 kb in size. Each minicircle codes for three to five gRNAs (Hajduk and Ochsenreiter, 2010; Hong and Simpson, 2003; Ochsenreiter et al., 2007). A prophase *T. brucei* cell typically contains a single mitochondrion with one kDNA kinetoplast. kDNA is made up of 5000 minicircles with several hundred different minicircle classes, and 25 maxicircles, which are virtually identical. Each minicircle is physically connected to three other minicircles and the maxicircles are interwoven into the minicircle network (Chen et al., 1995). Overall, the kDNA resembles a chain mail and is likely organized through several histone-like proteins (Lukeš et al., 2001; Xu et al., 1996). In *T. brucei*, the whole network builds a disc-like structure of ∼450 nm in diameter and 150 nm in height (Jakob et al., 2016). The replication of the kDNA is complex, like its structure, and some estimate that up to 150 proteins are involved in this process (Jensen and Englund, 2012). The current replication model suggests that the minicircles are released in a covalently closed (CC) conformation from the network into the kinetoflagellar zone (KFZ), the region between the kinetoplast and the inner mitochondrial membrane (Drew and Englund, 2001; Jensen and Englund, 2012). Here, replication is initiated, proceeds unidirectionally via theta intermediates and forms two nicked/gapped (N/G) minicircles that are then transported to the antipodal sites via an unknown mechanism (Jensen and Englund, 2012; Povelones, 2014). The antipodal sites are ill-defined protein complexes at opposing sites of the kDNA disc. Within these sites, partial gap repair occurs and the minicircles are reattached to the kDNA network by a topoisomerase type II enzyme (Wang et al., 2000). The newly replicated and reattached minicircles maintain at least one nick/gap each until the networks are separated. The repair of the nicks and gaps is likely mediated by the mitochondrial ligase LigKα (Downey et al., 2005) and the mitochondrial polymerase Polβ PAK (Saxowsky et al., 2003). Subsequently, the duplicated kDNA is segregated through the separating basal bodies that are connected to the kDNA via the TAC structure (Ogbadoyi, 2003). Several components of these three regions of the TAC have now been identified: p197 and TAC65 in the exclusion zone filaments (EZFs) (Gheiratmand et al., 2013; Käser et al., 2016), TAC40, TAC42 and TAC60 in the outer mitochondrial membrane (Käser et al., 2017; Schnarwiler et al., 2014), p166 at the inner mitochondrial membrane (Zhao et al., 2008) and TAC102 in the unilateral filaments (ULFs) (Trikin et al., 2016). In a recent study, we elucidated the hierarchy of TAC biogenesis suggesting a *de novo* assembly of the complex (Hoffmann et al., 2018). Furthermore, we show that, of the identified TAC proteins, TAC102 is the most proximal to the kDNA, while p197 is most distal to the kDNA (Hoffmann et al., 2018).

Here, we present data that characterizes the minicircle replication factor 172 (MiRF172; Tb927.3.2050) as a kDNA-associated protein essential for normal growth and kDNA maintenance in *T. brucei*. We also demonstrate that MiRF172 is likely involved in the reattachment of replicated minicircles to the kDNA disc. Furthermore, we demonstrate that localization of MiRF172 depends on the kDNA and partially on the TAC, which suggests that there is an interaction between replication and segregation machinery.

## RESULTS

**Fig. 1.**
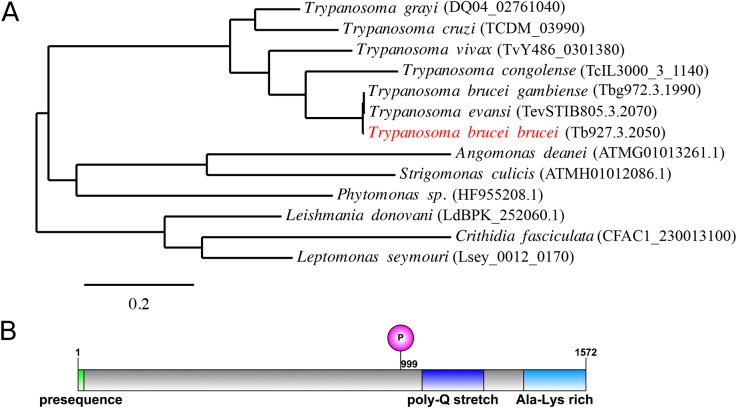
**Phylogeny and protein domains of MiRF172.** (A) A phylogenic tree showing the conservation of MiRF172 among Kinetoplastids. The position of MiRF172 in *T. brucei brucei* is highlighted in red. The scale bar indicates the number of amino acid substitutions. (B) Illustration of the MiRF172 ORF. Depicted are, in green, the mitochondrial-targeting sequence, in magenta, the phosphorylation site at position 999, in dark blue, the poly-Q stretch enriched domain, and in light blue, the alanine and lysine-enriched C-terminal domain.

MiRF172 is a very basic (pI 9.5), large (172 kDa), conserved hypothetical protein (Fig. 1A) with a predicted mitochondrial-targeting sequence at the N-terminus (Fig. 1B). MiRF172 has been detected in several proteomics studies that (1) support it having mitochondrial localization (Peikert et al., 2017; Zhang et al., 2010), (2) show that it is developmentally regulated (Gunasekera et al., 2012) and (3) indicate a possible phosphorylation site at S999 (Urbaniak et al., 2013). The gene, including its position in the genome, is conserved throughout the currently sequenced Kinetoplastea (Fig. 1A). The protein contains a poly-Q stretch and an alanine-lysine-rich region, both of which are found in the C-terminal part (Fig. 1B). While the poly-Q stretch is only conserved among the *Trypanosoma* species, the alanine-lysine-rich region is conserved throughout the Kinetoplastea.

### MiRF172 protein localizes to the kDNA

**Fig. 2.**
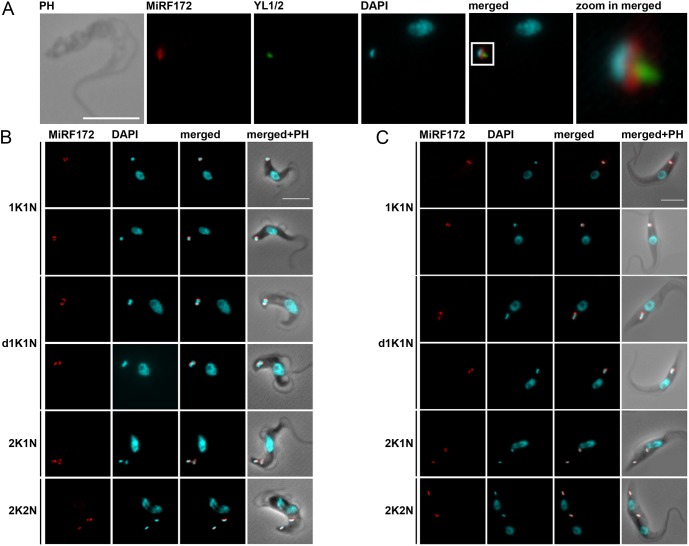
**Localization of MiRF172 in BSF and PCF *T. brucei* cells.** (A) Immunofluorescence microscopy of MiRF172–PTP-expressing BSF cells. The localization of MiRF172–PTP (red) is represented by maximum intensity projections from immunofluorescence microscopy image stacks of *T. brucei* BSF cells. MiRF172–PTP was detected by means of the anti-Protein A antibody. The mature basal bodies were detected with the YL1/2 monoclonal antibody (green). The kDNA and the nucleus were stained with DAPI (cyan). (B) Immunofluorescence analysis of MiRF172–PTP during different stages of the cell cycle (1K1N, dK1N, 2K1N, 2K2N) in BSF cells. K, kDNA; N, nucleus; dK, duplicating kDNA. Localization of MiRF172–PTP (red) and DNA (cyan) were performed as described in A. (C) Immunofluorescence analysis of MiRF172–HA during different stages of the cell cycle (1K1N, dK1N, 2K1N, 2K2N) in PCF cells. The localization of MiRF172–HA (red) is represented by maximum intensity projections from immunofluorescence microscopy image stacks of PCF cells. MiRF172–HA was detected with anti-HA antibody. The kDNA and the nucleus were stained as described in A. PH, phase contrast. Scale bars: 5 µm.

**Fig. 3.**
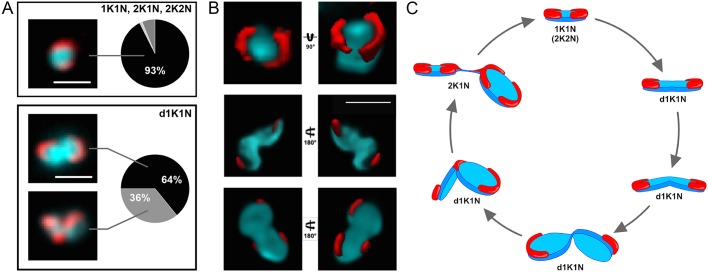
**Analysis of MiRF172 localization during the cell cycle.** (A) Quantification of MiRF172–PTP localization at single or duplicated kDNA discs (1K1N, 2K1N, 2K2N) and duplicating kDNA discs (d1K1N) in BSF cells (n_total_=240). K, kDNA; dK, duplicating kDNA; N, nucleus. The left side shows representative immunofluorescence microscopy images depicting the localization of MiRF172–PTP (red) relative to the kDNA disc (cyan). The pie charts show the percentage localization of MiRF172 in the respective kDNA replication stage. (B) 3D-STED immunofluorescence analysis of MiRF172–PTP in *T. brucei* BSF cells. An MiRF172 (red) and kDNA (cyan) 3D projection (surface rendering) from different angles is shown. MiRF172-PTP was detected with the anti-Protein A antibody and images acquired by 3D-STED microscopy. The kDNA was stained with DAPI (cyan) and images acquired by confocal microscopy. Pictures were deconvolved with the Huygens professional software. (C) Model of MiRF172 localization during the cell cycle. Depicted is a model of the different stages of kDNA disc (cyan) replication in *T. brucei* and the localization of MiRF172 (red) relative to the kDNA. Scale bars: 1 µm.

To localize the MiRF172 protein, we tagged it *in situ* at the C-terminus with a PTP epitope tag in bloodstream form (BSF) and with HA in procyclic form (PCF) *T. brucei* (Fig. 2). Based on colocalization studies in BSF cells with the basal body marker YL1/2 and the DNA stain DAPI, the protein localizes at the kDNA, facing the KFZ (Fig. 2A). MiRF172 is expressed throughout the cell cycle in both life cycle stages (Fig. 2B,C). The protein forms two foci that are 180° apart on kDNA discs in the vast majority of the cells [93% of 1K1N, 2K1N and 2K2N cells; where K is kinetoplast, N is nucleus (hence, 1K1N cells are in G1 of the cell cycle, 2K1N cells are in nuclear G2 phase, and 2K2N cells exist just prior to cytokinesis)] (Fig. 2B,C; Fig. 3A, upper panel). The position on the kDNA disc is reminiscent of the antipodal sites that have been described for many kDNA-associated proteins (Jensen and Englund, 2012). In rare cases, we also observed localization of MiRF172 covering the whole disc (1% of 1K1N, 2K1N or 2K2N cells) or surrounding the entire kDNA disc (6% of 1K1N, 2K1N or 2K2N cells). When the mitochondrial genome adopts a bilobed structure during kDNA replication (Fig. 2B,C, d1K1N) MiRF172 remains as two foci on the opposing sides (64% of d1K1N cells, Fig. 3A lower panel) until to just prior to when the kDNA divides. That is when a third spot appears in the middle between the two segregating discs (36% of all d1K1N cells, Fig. 3A, lower panel, Fig. 3C). After kDNA segregation, the second spot is present on each of the kDNA discs (Fig. 3C; 2K1N). In 3D reconstructions of 1K1N kDNA discs from super-resolution stimulated emission depletion (STED) microscopy images, the protein forms two curved structures each covering ∼25% of the kDNA circumference facing the KFZ (Fig. 3B).

We used detergent extraction with digitonin to isolate mitochondria as described previously (Trikin et al., 2016). Solubilization of the mitochondrial fraction with 1% digitonin leads to an insoluble and soluble fraction (Foucher et al., 2006). MiRF172 remained mostly associated with the insoluble fraction even after DNaseI treatment (Fig. S1A–C). Furthermore, we isolated flagella as described previously (Ogbadoyi, 2003) and could show that MiRF172 remains associated with flagella (Fig. S1D), similar to what has been described for TAC components (Käser et al., 2016, 2017; Schnarwiler et al., 2014; Trikin et al., 2016; Zhao et al., 2008).

### RNAi-mediated depletion of MiRF172 leads to growth retardation and kDNA loss

**Fig. 4.**
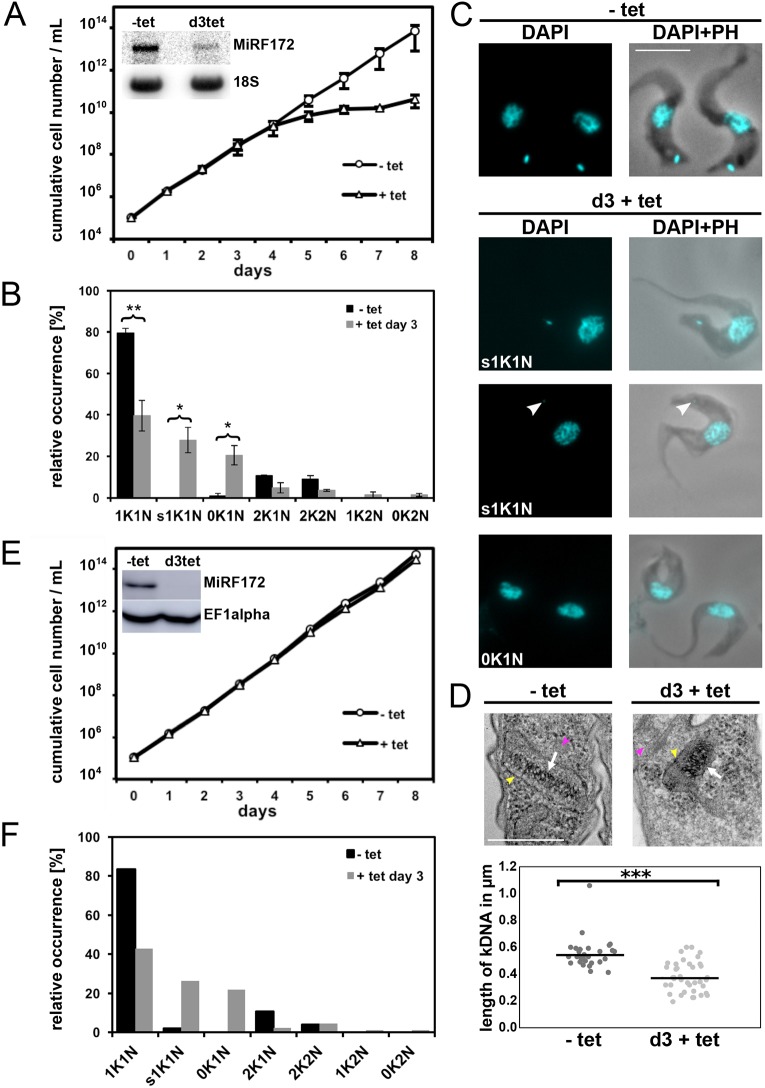
**Phenotype upon knockdown of MiRF172 mRNA by RNAi in *T. brucei* BSF cells.** (A) Growth curve of *T. brucei* BSF cells expressing MiRF172 RNAi. Results are mean±s.d. The inset depicts a northern blot, showing ablation of MiRF172 mRNA at day 3 (d3) post induction. 18S rRNA serves as a loading control. (B) Quantification of the relative occurrence of kDNA discs and nuclei in MiRF172 RNAi induced (+tet day 3) and uninduced cells (−tet) (*n*≥100 for each condition). K, kDNA; N, nucleus; sK, small kDNA. Results are mean±s.d. *P<0.05, **P≤0.01 (unpaired two-tailed *t*-test). (C) Representative fluorescence microscopy images of MiRF172 RNAi BSF cells. The nucleus and the kDNA were stained with DAPI. Arrowheads point to small kDNA. PH, phase contrast. Scale bar: 5 µm. (D) Upper panels, representative images of the ultra-structures of the kDNA of MiRF172 RNAi cells revealed by TEM. White arrows point to the kDNA, yellow arrowheads to the mitochondrial membrane, magenta arrowheads to basal body or base of flagellum. Scale bar: 500 nm. Lower panel, length measurements (*n*≥30 for each condition) of kDNA ultra-structures from uninduced (mean length=544 nm) and induced (3 days; mean length=368 nm) MiRF172 RNAi BSF cells. ***P≤0.001 (unpaired two-tailed *t*-test). (E) Growth curve of MiRF172 RNAi BSF γL262P *T. brucei* cells. The inset depicts a western blot showing ablation of MiRF172–PTP protein at day 3 post induction. EF1α serves as a loading control. (F) Quantification of the relative occurrence of kDNA discs and nuclei in MiRF172 RNAi γL262P *T. brucei* cells (*n*≥100 for each condition).

To study the function of MiRF172 we depleted the mRNA by RNAi in NYsm BSF cells using the tetracycline (tet) inducible RNAi vector pTrypRNAiGate. Northern blot analysis showed a decrease of MiRF172 mRNA by 68% on day 3 of induction (Fig. 4A, inset). After RNAi induction, cells grow at normal rates until day 4, when a growth defect becomes visible that is maintained at least until day 8. The growth defect was not accompanied by any obvious change in cell morphology or motility. In order to characterize a potential effect of MiRF172 depletion on mitochondrial genome replication, we sampled the population (*n*≥100 for each condition) at day 0 and 3 post induction. We stained the cells with the DNA dye DAPI and evaluated the relative occurrence of kDNA discs and nuclei in different cell cycle stages: cells with one kDNA disc and one nucleus (1K1N; cells are in G1 of the cell cycle), cells with already replicated and segregated kDNA discs and one nucleus (2K1N, cells are in nuclear G2 phase), as well as cells that had replicated both the kDNA and the nucleus (2K2N, cells just prior to cytokinesis). We also screened for any abnormal K-N combinations like 1K2N (likely a product of kDNA missegregation), 1K0N (zoid cells) as well as 0K1N (indicative of kDNA replication/segregation defects). The major change in K-N combinations was the accumulation of 0K1N cells to ∼20% at day 3 post induction, just prior to the appearance of the growth phenotype (Fig. 4B,C). At the same time point, we observed that 30% of 1K1N cells had smaller kDNA discs (s1K1N) compared to uninduced cells (Fig. 4B,C). To test whether the observed changes were significant, we performed the experiment in triplicates.

### RNAi-mediated depletion of MiRF172 leads to an accumulation of smaller kDNA networks

To better characterize the kDNA loss phenotype, we performed transmission electron microscopy (TEM) on ultrathin sections (70–80 nm) and examined the ultra-structure of the kDNA networks of BSF cells induced for RNAi against MiRF172 and compared them to uninduced cells. We measured the length of the striated structure, which corresponds to a cross section through the kDNA disc, in randomly acquired kDNA images from uninduced (*n*=30) and induced cells (*n*=38). In uninduced cells, the mean diameter of the kDNA was 544 nm, while in induced cells it was significantly (*P*≤0.001) reduced to 368 nm (Fig. 4D; Fig. S2). Although the size (diameter) of the network was reduced, the overall appearance of the striated structure and its relative position to the basal body did not change (Fig. 4D; Fig. S2). To validate the appearance of smaller kDNA discs in electron microscopy, we also measured DAPI stained networks from epifluorescence microscopy images of MiRF172 RNAi-induced cells. After 3 days of MiRF172 depletion, the size of the networks (*n*≥297 for each condition) was significantly decreased (*P*≤0.0001) to ∼70% of the size in the uninduced cells (Fig. S3).

### RNAi-mediated depletion of MiRF172 in γL262P BSF cells has no influence on growth

In order to test whether MiRF172 has essential functions that are not directly related to kDNA maintenance and whether the kDNA loss phenotype is a secondary effect, we used the γL262P cell line, which harbors a single point mutation in the F_1_F_0_-ATPase and is able to compensate for the loss of the kinetoplast in a way that is similar to ‘petite’ mutants in yeast (Dean et al., 2013). γL262P BSF cells were transfected with the inducible RNAi vector, which was previously used to generate the MiRF172 RNAi BSF cell line. We essentially performed the same analysis as described above and observed that, while the γL262P MiRF172 RNAi cells lost kDNA at a similar rate (*n*≥100 for each condition) as the NYsm strain, the cells showed no additional growth phenotype, suggesting that the sole function of MiRF172 is in kDNA maintenance and loss of kDNA is a direct effect of the depletion of MiRF172 (Fig. 4E,F).

### RNAi-mediated depletion of MiRF172 leads to a loss of mini- and maxicircles

**Fig. 5.**
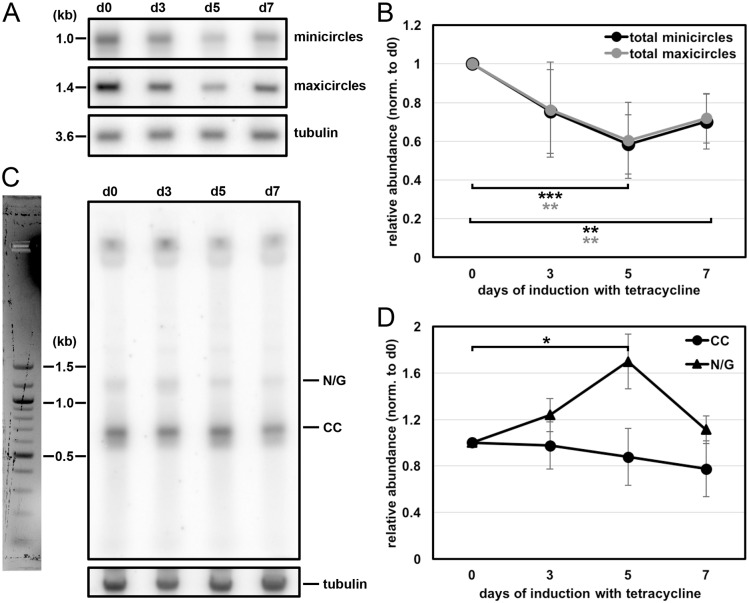
**Effect of MiRF172 RNAi on the kDNA abundance and free minicircle replication intermediates in *T. brucei* BSF cells.** (A) Detection of total minicircles and maxicircles by Southern blotting. Total DNA, digested with HindIII and XbaI, from either uninduced [day (d)0] or RNAi induced (d3, d5, d7) cells was used. This was probed for minicircles (detection of 1.0-kb linearized minicircles), maxicircles (detection of a 1.4-kb fragment), and tubulin, as a loading control (detection of a 3.6-kb fragment). (B) Quantification of minicircle and maxicircle abundance on Southern blots during MiRF172 depletion (*n*≥6 for each time point). Black, minicircles; gray, maxicircles. The ratio of the abundance of total minicircle or maxicircle relative to the loading control (tubulin), normalized to day 0 of RNAi induction, is shown. **P≤0.01, ***P≤0.001 (gray values are for minicircles, and black for maxicircles). (C) Detection of free minicircle replication intermediates by Southern blotting. Total DNA isolated from either uninduced (d0) or RNAi induced (d3, d5, d7) cells was Southern blotted, and probed for minicircles. N/G and CC minicircles are indicated. Tubulin was used as a loading control. (D) Quantification of CC and N/G minicircles, as determined by Southern blotting, during MiRF172 depletion (*n*≥6 for each time point). Black circles, CC minicircles, black triangles, N/G minicircles. The ratio of the abundance of minicircles relative to the loading control tubulin and normalized to day 0 of RNAi induction is shown. *P<0.05.

The results described above suggest that MiRF172 is involved in kDNA replication. To study the effect of kDNA loss in more detail, we performed Southern blot analyses of minicircles and maxicircles in MiRF172 RNAi BSF cells. Whole-cell DNA was extracted at day 0, 3, 5 and 7 post RNAi induction. For each time point, we included at least six biological replicates. To analyze total amount of minicircles and maxicircles, we used DNA samples that were digested with HindIII and XbaI. As a loading control, we used a probe targeting the tubulin intergenic region. We detected a decrease of minicircle and maxicircle abundance to ∼60% of the uninduced levels from day 0 to day 5 post induction of RNAi after which the amount of minicircles and maxicircles increased again to ∼70% of the uninduced levels (Fig. 5A,B). To further study the effect of MiRF172 depletion on minicircle replication, we performed Southern blot analysis of minicircles released from the network, prior to (CC) and post replication (N/G). For this, undigested DNA from uninduced and MiRF172 depleted cells at day 3, 5 and 7 post MiRF172 RNAi induction was used. We detected a steady decrease of the CC minicircles that had been released from the network but had not yet been replicated (Fig. 5C,D). However, the N/G minicircle population, representing the newly replicated intermediates prior to reattachment to the disc, increased significantly (*P*<0.05) until day 5 post RNAi induction. They then returned to the initial levels (Fig. 5C,D). Based on these results, we suggest that MiRF172 is involved in the replication of the kDNA and more specifically in the process for reattachment of the replicated minicircles to the kDNA network.

### The TAC is required for proper MiRF172 localization

**Fig. 6.**
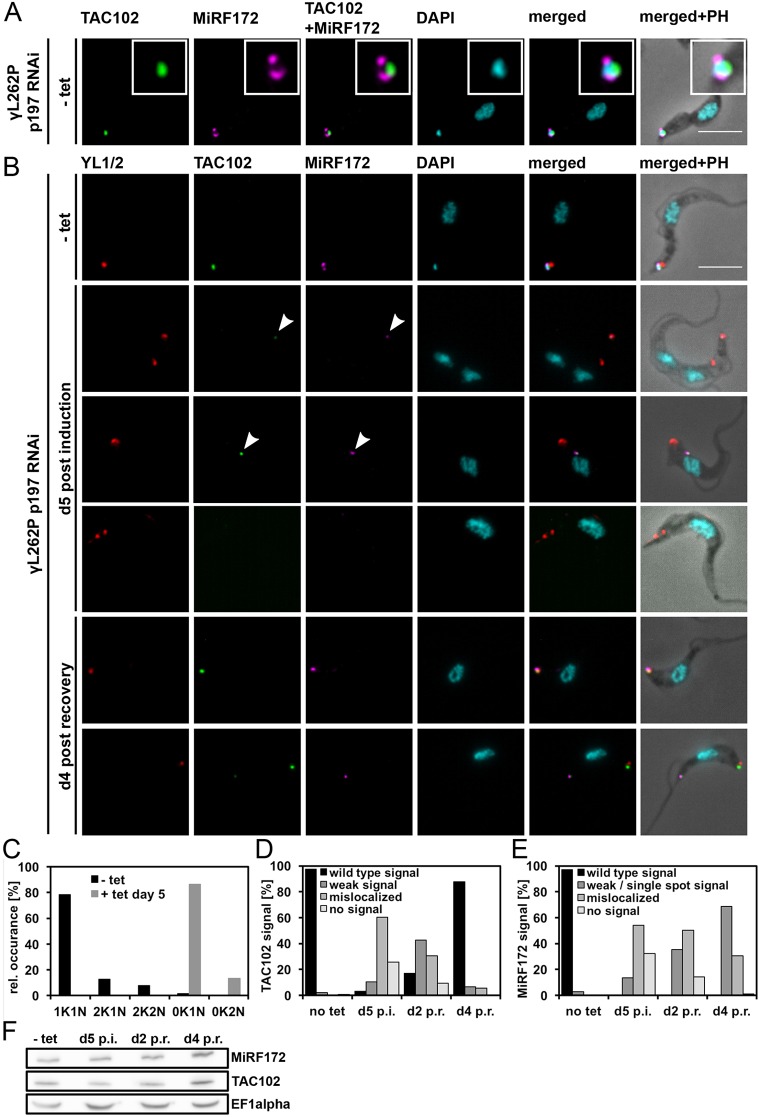
**MiRF172 and TAC102 after p197 RNAi depletion and recovery after removal of tet in γL262P p197 RNAi BSF *T. brucei* cells.** (A) Colocalization of MiRF172–PTP with TAC102 in γL262P p197 RNAi BSF cells. Localization of MiRF172–PTP (magenta) and TAC102 (green) is represented by maximum intensity projections from immunofluorescence microscopy image stacks of γL262P p197 RNAi BSF *T. brucei* cells. MiRF172–PTP was detected with anti-Protein A antibody. TAC102 was detected with anti-TAC102 monoclonal mouse antibody. The kDNA and the nucleus were stained with DAPI (cyan). The inset shows a higher magnification view. (B) TAC recovery experiment in γL262P p197 RNAi BSF *T. brucei* cells. To detect MiRF172–PTP, TAC102 and DNA the same antibodies and reagents as in A were used. The pictures were obtained under the same conditions as in A. The basal bodies (red) were detected with the YL1/2 monoclonal antibody. - tet, uninduced cells; d5 post induction, MiRF172-depleted cells at day 5 of RNAi (RNAi was induced by addition of tet); d4 post recovery, after 5 days of RNAi, tet was removed and cells were grown for 4 additional days. (C) Quantification of the relative occurrence of kDNA discs and nuclei in γL262P p197 RNAi induced and uninduced cells (*n*≥113 for each time point). K, kDNA; N, nucleus. (D) Quantitative analysis of TAC102 in γL262P p197 RNAi cells without tet (no tet), with tet at day five (d5 p.i.) as well as 2 days after removal of tet (post recovery; d2 p.r.) and at day 4 post recovery (d4 p.r.) (*n*≥105 for each time point). (E) Quantitative analysis of the MiRF172–PTP signal in in γL262P p197 RNAi cells as in D (*n*≥105 for each time point). (F) Western blot analysis of γL262P p197 RNAi BSF cells. Total protein isolated from uninduced cells (−tet), cells induced with tet for 5 days (d5 p.i.) and cells released from p197 RNAi at day 2 (d2 p.r.) and day 4 post recovery (d4 p.r.) was used. C-terminally PTP-tagged MiRF172 was detected with the anti-PAP antibody and TAC102 with the anti-TAC102 monoclonal mouse antibody. EF1α serves as a loading control. Arrowheads point to the TAC102 and MiRF172 signals. PH, phase contrast. Scale bars: 5 µm.

Based on the observation that MiRF172 remains associated with the flagellum after flagellar extraction from BSF cells (as occurs with TAC102; Fig. S1D) and that MiRF172 localizes at the kDNA disc sides facing the KFZ, a region that overlaps with the ULFs (Fig. 2A), we wondered whether the protein would colocalize with a TAC marker protein of the ULFs, such as TAC102. To determine this, we used immunofluorescence microscopy. The images show that MiRF172 is located between TAC102 and the kDNA disc, with little to no overlap between the MiRF172 and the TAC102 signal (Fig. 6A).

Based on proximity of the TAC and MiRF172, we wanted to test whether the TAC itself has an impact on the localization of MiRF172. For this, we created a γL262P BSF cell line expressing tagged MiRF172 that allows inducible depletion of p197, a TAC component in the EZFs (Gheiratmand et al., 2013). Loss of the p197 protein leads to disruption of the TAC connection between the basal bodies and the kDNA, loss of the kDNA and mislocalization of all currently known TAC components, including TAC102, as described previously (Hoffmann et al., 2018). Through epifluorescence microscopy, we could replicate the kDNA- and TAC102-loss phenotype (Fig. 6B–D) and additionally observed MiRF172 mislocalization (54%) or loss of detection (32%) after 5 days of p197 depletion (Fig. 6B,E). While MiRF172 protein is mislocalized or not detectable through immunofluorescence microscopy, it remains unchanged in its expression level as detected by western blotting (Fig. 6F). After re-expression of p197 for 4 days almost all cells showed a wild-type signal for TAC102 in epifluorescence microscopy (Fig. 6D). However, we could not observe any cell with the wild-type localization for MiRF172 (Fig. 6E). At day 4 post recovery, the majority of cells (69%) expressed MiRF172 but in a single spot close to TAC102 and the basal body, while ∼30% of the cells showed a mislocalized expression of MiRF172 (Fig. 6B,E). When we checked for cells that contained a replicated and matured basal body using the YL1/2 antibody, we found a TAC102 signal associated with both basal bodies. However, the signal for MiRF172 was exclusively detected in proximity to the ‘old’ basal body/TAC that is found in the more anterior position (Fig. S4).

### RNAi-mediated depletion of MiRF172 does not lead to loss of the TAC

**Fig. 7.**
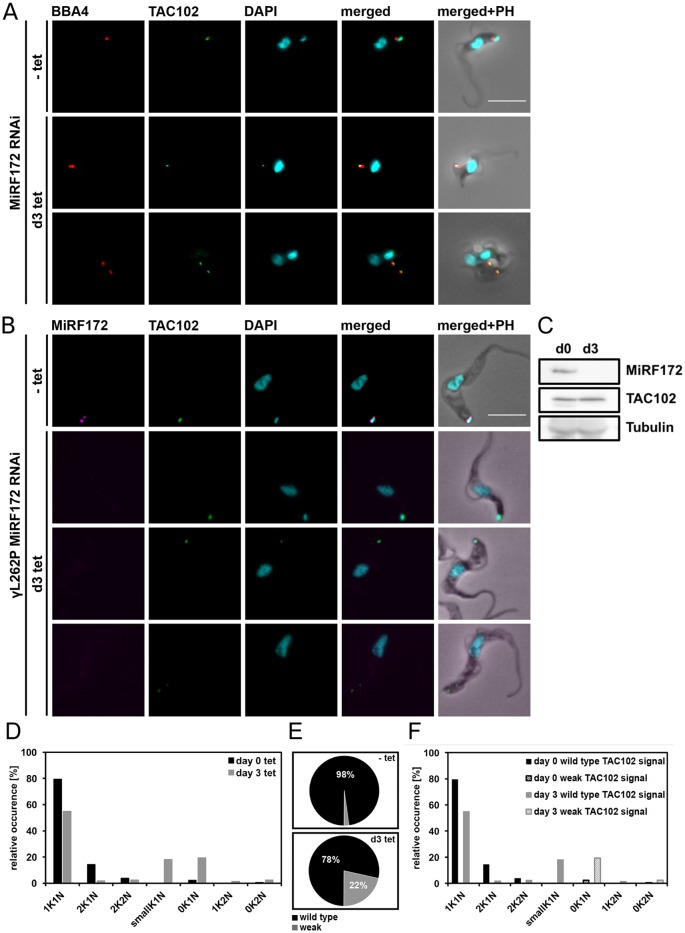
**Quantification of TAC102 in MiRF172 RNAi BSF cells.** (A) MiRF172 RNAi BSF cells stained for TAC102 (green) and basal bodies (red) from either uninduced (-tet) or RNAi induced [day (d)3] cells. Pictures show maximum intensity projections from immunofluorescence microscopy image stacks of MiRF172 RNAi BSF *T. brucei* cells. TAC102 was detected with the anti-TAC102 polyclonal rat antibody and the basal bodies with the monoclonal mouse antibody BBA4. The kDNA and the nucleus were stained with DAPI (cyan). (B) γL262P MiF172 RNAi BSF cells stained for MiRF172–PTP (magenta), TAC102 (green), basal bodies (red) and DAPI (cyan). Proteins and DNA were detected with the same antibodies and reagents as in A. MiRF172–PTP was detected with the anti-Protein A antibody. The pictures show maximum intensity projections as in A. (C) Western blot analysis of γL262P MiRF172 RNAi BSF cells. Total protein isolated from uninduced cells (d0) and cells induced with tet for 3 days (d3). C-terminally PTP-tagged MiRF172 was detected with an anti-PAP antibody and TAC102 with the anti-TAC102 monoclonal mouse antibody. Tubulin serves as a loading control. (D) Quantification of the relative occurrence of kDNA discs and nuclei in γL262P MiRF172 RNAi induced and uninduced cells (*n*≥180 for each time point). K, kDNA; N, nucleus. (E) Quantification of TAC102 in γL262P MiRF172 RNAi uninduced (−tet) and cells induced for three days with tet (d3 tet). Black represents the wild-type TAC102 signal and gray stands for a weak TAC102 signal. (F) Quantification of the relative occurrence of the TAC102 signal in γL262P MiRF172 RNAi cells with different kDNA and nucleus DNA content. PH, phase contrast. Scale bars: 5 µm.

As mentioned above, biogenesis of MiRF172 at the kDNA disc occurs during kDNA division (Fig. 3C) and thus prior to the replication of the TAC102 signal (Trikin et al., 2016). To test whether depletion of MiRF172 has an impact on TAC biogenesis, we probed for TAC102, the currently kDNA most proximal component of the TAC, during three days of MiRF172 RNAi (Fig. 7A–C). We detected the efficient depletion of MiRF172 (Fig. 7B,C) and some changes in the TAC102 signal (Fig. 7A,B), whereas we did not detect changes of TAC102 abundance by western blot analysis (Fig. 7C). In order to observe MiRF172 depletion we had to resort to the γL262P cell line since we were unable to create an MiRF172 RNAi and tagged cell line in our wild-type strain (Fig. 6B). Depletion of MiRF172 in the γL262P cell line expressing a tagged MiRF172-PTP led to the same phenotype as described above (Figs 4 and 7D). As determined by epifluorescence microscopy, the signal for TAC102 remained unchanged in the majority of the cells (78%) but became weaker in the 0K1N cells (22%) (Fig. 7E,F). However, we never detected a complete loss of TAC102 in any of the MiRF172-depleted cells.

## DISCUSSION

MiRF172 shares overall no similarities with other proteins, except for the low complexity regions of the C-terminus (Fig. 1), which shows weak similarities to a transcription corepressor (trfA) and a putative kinase from *Dictyostelium* (Aslett et al., 2009). Consistent with its essential function in kDNA replication, MiRF172 is conserved in the currently sequenced Kinetoplastea (Fig. 1A). Of the two recognizable domains (Fig. 1B), the C-terminal alanine-lysine-rich region is present in all currently sequenced Kinetoplastea, while the poly-Q stretch is only found in the genus *Trypanosoma*. We speculate that this variation in the MiRF172 sequence is related to the two kDNA replication models, which have been proposed for *Crithidia* and *T. brucei*, which essentially differ in the reattachment mechanism (Jensen and Englund, 2012)*.* MiRF172 localizes to two regions around the mitochondrial genome, which have been described as the antipodal sites in numerous publications (Jensen and Englund, 2012; Povelones, 2014). However, the actual composition and dynamics of the antipodal sites during the kDNA replication cycle, as well as the relative position of the individual components in this large structure, remain mostly unknown. MiRF172 is present throughout the entire mitochondrial genome replication and segregation phase in *T. brucei*, similar to the primases PRI1 and PRI2, the helicases Pif1 and Pif5, the endonuclease SSE1 and the mitochondrial type II topoisomerase (Engel and Ray, 1999; Hines and Ray, 2010, 2011; Li et al., 2007; Liu et al., 2006, 2010). The polymerase Polβ, in contrast, is only detected at the antipodal sites during replication (Bruhn et al., 2010; Saxowsky et al., 2003). We describe MiRF172 as a novel mitochondrial genome replication factor in *T. brucei*. The current model predicts that replication of the minicircles is initiated after the release into the KFZ by a topoisomerase activity, through binding of the protein UMSBP and several replication factors, including the two polymerases PolIB and PolIC (Bruhn et al., 2010, 2011; Milman et al., 2007). The minicircles are then moved to the antipodal sites by an unknown mechanism. At the antipodal sites, primers are removed by the single-strand endonuclease SSE-1 and the helicase Pif5, after which gaps are filled by polymerase Polβ and finally the ligase LigKβ seals most of the gaps (Downey et al., 2005; Engel and Ray, 1999; Klingbeil et al., 2002; Saxowsky et al., 2003). Afterwards, the minicircles reattach to the growing network, likely because of topoisomerase activity (Wang and Englund, 2001). In the kDNA disc, the last minicircle gaps are repaired through a combination of Polβ–Pak and the DNA ligase LigKα and likely other proteins (Downey et al., 2005; Klingbeil and Englund, 2004; Klingbeil et al., 2002). Based on the current model, an accumulation of gapped free minicircles as detected in the MiRF172 RNAi analysis, points towards a function of MiRF172 in the reattachment process. This is similar to the RNAi phenotype of the topoisomerase II that also leads to an increase of the N/G minicircles, while the abundance of CC minicircles initially change very little (Wang and Englund, 2001). Topoisomerase II has been localized to the antipodal sites (Wang and Englund, 2001) and since MiRF172 localization potentially overlaps with the antipodal sites, we predict that MiRF172 interacts with topoisomerase II in the process of minicircle reattachment in *T. brucei*. One could imagine that MiRF172 might aid the topoisomerase in the discrimination between replicated and non-replicated minicircles. In the future, biochemical co-immunoprecipitation studies should enable us to test this model. While the depletion of MiRF172 clearly led to smaller kDNAs (Fig. 4B,C), it did not change the ultrastructure of the disc (Fig. 4D, Fig. S2) supporting its role in attachment of the minicircles rather than modulation of the disc. Although we carefully analyzed the TEM images, we cannot exclude that the size difference we detected was based on a biased selection of images towards larger discs in the wild-type cells and thus we also measured kDNA disc size changes by epifluorescence microscopy (Fig. S3), which is much less likely to lead to a bias in image selection. With the data from epifluorescence images we were able to confirm the TEM results.

During the RNAi experiments in the NYsm strain, we never achieved depletion of MiRF172 RNA below the level of detection (Fig. 4A, inset), and we think that the mild phenotype where there is only a decrease of 30–40% in minicircle and maxicircle abundance is a consequence of the residual MiRF172. The increase in minicircle and maxicircles between 5 and 7 days post induction of MiRF172 RNAi is likely due to a loss of RNAi function, a phenomenon often observed in *T. brucei*.

The proximity of the kDNA replication and segregation machinery in *T. brucei* could suggest a physical interaction of the components involved in the two processes in the KFZ. This is supported by the biochemical fractionations indicating that MiRF172 remains associated with the isolated flagellum (as is the TAC, Fig. S1D) and is present in the pellet fraction of the digitonin extraction even after DNaseI treatment (Fig. S1A–C). Furthermore, the TAC seems to provide some information for the localization of MiRF172 since in the ‘petite’ trypanosomes MiRF172 relocalizes to the TAC region even in the absence of kDNA (Fig. 6). Interestingly, the localization information seems to be exclusively at the ‘old’ basal body TAC structure, while the new TAC is unable to direct MiRF172 localization in these cells (Fig. S4). One explanation for this is that the signal for MiRF172 localization is transferred from the ‘old’ TAC to the new developing TAC via the kDNA (also see model in Fig. 3C) such that if the kDNA is missing, the transfer is interrupted and only after cell division will the TAC be competent to provide the localization information for MiRF172.

Although TAC102 was always detected at the correct position even in the absence of MiRF172 (Fig. 7), we noticed that the signal for TAC102 was weaker when the kDNA was completely lost (0K1N, Fig. 7E,F), while this was not detected in the p197 RNAi recovery experiments (Fig. 6B,D). This suggests that the combination of MiRF172 and kDNA loss interferes with TAC102 biogenesis.

Overall, we describe a novel kDNA replication factor possibly involved in reattachment of minicircles to the kDNA disc.

## MATERIALS AND METHODS

### *T. brucei* cell culture conditions

Monomorphic *T. brucei* BSF NYsm (Wirtz et al., 1999) and NYsm-derived γL262P (Dean et al., 2013) cells were cultured in Hirumi-modified Iscove's medium 9 (HMI-9) supplemented with 10% fetal calf serum (FCS) and incubated at 37°C and 5% CO_2_. PCF 427 *T. brucei* cells were cultured in semi-defined medium-79 (SDM-79) supplemented with 10% FCS at 27°C. Depending on the cell line, 5 μg/ml blasticidin, 2.5 μg/ml geneticin, 2.5 μg/ml hygromycin, 2.5 μg/ml phleomycin or 0.5 μg/ml puromycin were added to the medium. Expression of the RNAi construct was induced by adding tetracycline (tet) to a final concentration of 1 μg/ml. NYsm BSF, 427 PCF trypanosomes were obtained from the established collection of the Institute of Cell Biology, University of Bern, Bern, Switzerland. The γL262P strain of BSF cells is a kind gift of Achim Schnaufer (Institute of Immunology and Infection Research, University of Edinburgh, Edinburgh, UK) (Dean et al., 2013).

### Transfections of *T. brucei* cells

To obtain transgenic cell lines for both types of experiment (the knockdown phenotype or the localization of MiRF172), we transfected cells with the respective construct of interest. To integrate the construct of interest, we used homologous recombination mechanism of the cells. For transfections, 10 μg of linearized plasmid or PCR product were dissolved in 100 μl BSF transfection buffer (90 mM Na-phosphate, 5 mM KCl, 0.15 mM CaCl_2_, 50 mM HEPES pH 7.3) (Burkard et al., 2007). 4×10^7^ mid-log phase BSF cells were pelleted (1800 ***g*** for 8 min) and resuspended in 100 μl BSF transfection buffer containing the DNA. The cells were transferred into Amaxa Nucleofector cuvettes and transfections were conducted in the Amaxa Nucleofector II using program Z-001 (panel V 1.2 kV, panel T 2.5 kV, panel R 186 Ohm, panel C 25 μF). For transfections of PCF cells, 10 μg of PCR product were dissolved in 400 μl Zimmerman post-fusion medium (ZPFM; 132 mM NaCl, 8 mM KCl, 8 mM Na_2_HPO_4_, 1.5 mM KH_2_PO_4_, 0.5 mM MgAc_2_, 0.09 mM CaAc_2_). 10^8^ mid-log phase PCF cells were pelleted (1800 ***g*** for 8 min), resuspended in the ZPFM containing the DNA and transferred into Amaxa Nucleofector cuvettes. We conducted transfections at 1500 V, 180 Ohms and 25 μF (BTX). The transfected cells were left to recover for 20 h. Then, with appropriate antibiotics, we selected for correct integration of the construct.

### DNA constructs

We targeted the MiRF172 RNAi constructs against the open reading frame (ORF; 4409 to 4719 bp) and the 3′UTR (1 to 10 bp) of the gene Tb927.3.2050. Briefly, a PCR fragment with adaptor sequences was amplified from genomic DNA of NYsm BSF cells, and cloned in two steps into the pTrypRNAiGate vector by Gateway cloning (Kalidas et al., 2011) to generate the later hairpin loop double-stranded (ds)RNA for RNAi. The final plasmids were linearized with NotI HF (NEB) and used for transfection as described above. We induced the RNAi construct expression by treatment with tet. To tag MiRF172 at the C-terminus with PTP, we amplified the ORF positions 4404 to 4895 from genomic NYsm BSF cell DNA and cloned it between the ApaI and NotI sites of the pLEW100 based PTP tagging vector (Schimanski et al., 2005). We linearized the resulting plasmid with BsmI prior to transfection. For the C-terminal triple HA tagging, we performed a PCR with primers containing overhangs complementary to the ORF from bp 4617 to 4716 and the 3′UTR from bp 1 to 99. The pMOTagging plasmid served as a template (Oberholzer et al., 2006). Both tagging constructs were recombined into the endogenous locus to substitute for one of the Tb927.3.2050 alleles and thus were constantly expressed.

### Immunofluorescence analysis

To analyze the localization of MiRF172, TAC102 and basal body proteins, we used immunofluorescence analysis. For this, ∼10^6^ cells were spread onto a slide and fixed for 4 min with 4% paraformaldehyde (PFA) in PBS (137 mM NaCl, 2.7 mM KCl, 10 mM Na_2_HPO_4_, 2 mM KH_2_PO_4_ pH 7.4). After fixation and washing with PBS, cells were permeabilized with 0.2% Triton-X 100 in PBS for 5 min so that the antibodies could access their epitopes later. Then, cells were blocked with 4% bovine serum albumin (BSA) in PBS for 30 min. After blocking, slides were incubated for 45 or 60 min with the primary antibody followed by washing with PBS plus 0.1% Tween 20 (PBST) and incubation with the secondary antibody for 45 or 60 min followed by washing with PBST and PBS. All incubations were performed at room temperature. Primary and secondary antibodies were diluted in PBS plus 4% BSA as follows: rat-anti-HA (cat. no. ROAHAHA, Sigma), 1:1000; polyclonal rabbit anti-Protein A (cat. no. P3375, Sigma) detecting the PTP epitope, 1:2000; mouse antibody BBA4 (Woods et al., 1989) 1:100, rat antibody YL1/2 detecting tyrosinated tubulin as present in the basal body (Kilmartin et al., 1982), 1:100,000; monoclonal mouse anti-TAC102 antibody (Trikin et al., 2016), 1:2000; polyclonal rat anti-TAC102 antibody (Trikin et al., 2016), 1:5000; Alexa Fluor^®^ 594-conjugated goat anti-rabbit IgG (H+L) (Life Technologies), Alexa Fluor^®^ 488-conjugated goat anti-rabbit IgG (H+L) (Invitrogen); Alexa Fluor^®^ 488-conjugated goat-anti-rat IgG (H+L) (Life Technologies); Alexa Fluor^®^ 488-conjugated goat anti-mouse-IgG (H+L) (Invitrogen), Alexa Fluor^®^ 594-conjugated goat anti-mouse IgG (H+L) (Molecular Probes), Alexa Fluor^®^ 647-conjugated goat anti-rat IgG (H+L) (Life Technologies), all at 1:1000. Cells were mounted with ProLong^®^ Gold Antifade Mounting Medium with DAPI (Molecular Probes), which stains the DNA, and cover slips were added. Images were acquired with the Leica DM5500 B microscope (Leica Microsystems) with a 100× oil immersion phase contrast objective. Images were analyzed using LAS X software (Leica Microsystems) and ImageJ. Significance of the quantification of relative occurrence of kDNA discs and nuclei in different cell cycle stages was calculated by using an unpaired two-tailed *t*-test from three biological replicates.

### Super-resolution 3D STED microscopy

MiRF172-PTP BSF cells were used to analyze the MiRF172 localization at the kDNA in more detail with STED microscopy and, therefore, we spread cells onto a slide. Their fixation, permeabilization and blocking were performed as described above. Polyclonal rabbit anti-Protein A antibody (Sigma) and the Alexa Fluor^®^ 594-conjugated goat anti-rabbit IgG (H+L) antibody were used as described above. Cover glasses (Nr. 1.5, Marienfeld) suitable for 3D STED microscopy were used. Cells were mounted as described above. Images were acquired using the SP8 STED microscope (Leica, with a 100× oil immersion objective and the LAS X Leica software) as *z*-stacks with a *z*-step size of 120 nm. For the MiRF172-PTP signal, the 594 nm excitation laser and the 770 nm depletion laser were used. Owing to non-availability of suitable depletion laser, the DAPI signal was acquired with confocal settings. Images were deconvoluted with the Huygens professional software.

### TEM

Embedding of the cells and thin sectioning for TEM was performed as described previously (Trikin et al., 2016). Images of the ultrathin sections (70–80 nm) were obtained with an FEI Morgani electron microscope (Tungsten cathode). The microscope was equipped with a digital camera (Morada, 12 megapixels, Soft Imaging System) and the AnalySIS iTEM image analysis software. The kDNA structure of uninduced cells (−tet) and induced cells at day 3 upon induction (+tet d3) was measured using ImageJ. All images were taken at a magnification of 28,000×. Significance of the results was calculated by using an unpaired two-tailed *t*-test.

### SDS-PAGE and western blotting

Whole-cell lysates were used for western blot analysis. Cells were washed in PBS and resuspended in 1× Laemmli buffer (12 mM Tris-HCl pH 6.8, 0.4% SDS, 2% glycerol, 1% β-mercaptoethanol and 0.002% Bromophenol Blue) in PBS and incubated at 95°C for 5 min. Approximately 5×10^6^ cells were loaded onto a 4% or 6% gel, resolved and then blotted (Bio-Rad blotting system) onto PVDF Immobilon^®^-FL transfer membranes (0.45 μm, Millipore) for 1 h at 100 V. Membranes were blocked in PBST plus 5% skimmed milk powder. The rabbit peroxidase anti-peroxidase soluble complex (PAP) was diluted 1:2000 in PBST plus 5% skimmed milk and incubated for 30 min at room temperature. The mouse-anti-EF1α (cat. no. sc-21758, Santa Cruz Biotechnology), rat-anti-HA and the rabbit-anti-HA antibodies (cat. no. ROAHAHA Roche and N6908-2ML, respectively, Sigma) were used 1:1000 in PBST plus 5% skimmed milk. Secondary antibodies were: swine anti-rabbit-IgG conjugated to horseradish peroxidase (HRP) (1:10,000, Dako) and rabbit anti-rat HRP conjugate (1:10,000, Dako), all in PBST plus 5% skimmed milk. After each incubation with the antibodies, the membranes were washed three times for 5 min in PBST and once for 5 min in PBS. SuperSignal West Femto Maximum Sensitivity Substrate (Thermo Scientific) and Amersham™ Imager 600 (GE Healthcare Life Sciences) were used to visualize the protein bands on the blots.

### Northern blotting

Total RNA was extracted from mid-log phase MiRF172 RNAi BSF cells with 1 ml of RiboZol™ (Amresco) per 5×10^7^ cells. For northern blot analysis, 10 μg of total RNA was separated for 2 h at 100 V in a 1% agarose gel containing 6% formaldehyde. RNA was blotted onto Hybond nylon membranes with 20× SSC (3M NaCl and 0.3M Na-citrate, pH 7) by capillary transfer. The RNA on the nylon membrane was cross-linked with a Stratagene UV-Stratalinker. Membranes were pre-hybridized at 65°C for 1 h in hybridization solution [5× SSC, 1:12.5 100× Denhardt's solution (2% BSA, 2% polyvinylpyrrolidone, 2% Ficoll), 50 mM NaHPO_4_ pH 6.8, 1% SDS, 100 μg/ml salmon sperm DNA]. The sequence-specific probe for MiRF172 mRNA was generated by PCR (primers used were: 5′-ggggacaagtttgtacaaaaaagcaggctCCCTGAGAAGGAACTTGAGC-3′ and 5′-ggggaccactttgtacaagaaagctgggtGGCTGCTCATCTACCGCTT-3′, where lowercase letters indicate the sequence complementary to the plasmid used for cloning). The probes were denatured in ddH_2_O for 5 min at 95°C. The random primed DNA labeling kit (Roche) was used according to the manufacturer's manual to label the probes. For normalization, 1.8 μl 18S rRNA probes (10 μM; Trikin et al., 2016) was mixed with 12.5 μl H_2_O, 2.7 μl γ-^32^P-ATP (1 MBq), 2 μl PNK buffer (10×) and 1 μl T4 PNK and incubated for 30 min at 37°C. Reactions were stopped with 5 μl EDTA (0.2 M) and 75 μl TE buffer (1×) and incubated 5 min at 95°C. Probes were quenched for 2 min on ice, and 50 μl of probe mixture was added to the membrane. Probes and pre-hybridized membranes were incubated overnight at 65°C and washed in 2× SSC with 0.1% SDS and/or 0.2× SSC with 0.1% SDS at 60°C. Blots probed for MiRF172 RNA were exposed for 24 h to storage phosphor screens in metal cassettes (Amersham Bioscience) , while blots for 18S rRNA were exposed for 15 min only. After exposure the screens where scanned using a Storm PhosphoImager (Amersham Bioscience). ImageJ was used for image analysis and quantification.

### Southern blotting

Total DNA was isolated from mid-log phase MiRF172 RNAi BSF cells. For this, cells were washed in PBS and resuspended in 1 ml of phenol per 5×10^7^ cells. The experimental procedure and analysis was as described previously (Trikin et al., 2016). In brief, total DNA, either 5 or 10 μg undigested (for detection of free minicircles), or 5 μg digested with HindIII and XbaI (for detection of total minicircles and maxicircles) was resolved in 1% agarose gel in 0.5× TAE buffer for 35 min at 136 V for total minicircles, at 2 h at 135 V or 4 h at 50V for free minicircles. The DNA was blotted onto nylon membranes by capillary transfer with 20× SSC. For kDNA detection, we generated sequence-specific minicircle and maxicircle probes by PCR from total DNA of NYsm BSF *T. brucei*. For the minicircle probe, we amplified ∼100 bp of the conserved minicircle sequence (Trikin et al., 2016). The maxicircle probe was amplified from total DNA of NYsm BSF *T. brucei* too, by using the following primers: 5′-CTAACATACCCACATAAGACAG-3′ and 5′-ACACGACTCAATCAAAGCC-3′ (Liu et al., 2006). For the normalization, a tubulin probe (binding to the intergenic region between α- and β-tubulin, fragment size 3.6 kb; Trikin et al., 2016) was used; this was generated and labeled in the same way as the minicircle and maxicircle probes. Blots were exposed for 24, 48 or 72 h to storage phosphor screens in metal cassettes (Amersham Bioscience) and scanned with a Storm PhosphoImager (Amersham Bioscience). ImageJ was used for image analysis and quantification. Significance of the results was calculated with an unpaired two-tailed *t*-test.

### Digitonin fractionations

For digitonin fractionation, 10^7^ cells were collected and washed with PBS. The cells were then resuspended in SoTE buffer (0.6M sorbitol, 2 mM EDTA and 20 mM Tris-HCl, pH 7.5). Digitonin was added to a final concentration of 0.025% or 1%, and the mixture was incubated on ice for 5 min. When we treated the extractions with DNaseI, we used 2×10^7^ cells. In addition to the 1% digitonin, we also added DNaseI to the resuspended cells and incubated them on ice for 30 min instead of 5 min. To separate the fractions, cells were centrifuged at 8000 ***g*** for 5 min at 4°C. For western blot analysis, a cell equivalent of 10^7^ cells from both fractions (supernatant and pellet) was mixed with Laemmli buffer. For analysis of DNA content, a cell equivalent of 10^7^ cells from both fractions (supernatant and pellet) was mixed with 6× loading dye containing SDS from NEB.

### Flagellar extraction

For flagellar preparation (Ogbadoyi, 2003), EDTA was added to BSF cells in medium with a final concentration of 5 mM. Cells were washed with PBS and then resuspended in extraction buffer (10 mM NaH_2_PO_4_, 150 mM NaCl and 1 mM MgCl_2_) containing 0.5% Triton X-100, on ice. After one washing step with extraction buffer, cells were incubated on ice for 45 min in extraction buffer containing 1 mM CaCl_2_ and then subjected to immunofluorescence analysis as described above.

### kDNA size measurements in DAPI-stained cells

To measure the change of kDNA networks size upon RNAi of MiRF172, we spread ∼10^6^ uninduced or induced cells at day 3 after induction with tet onto slides, and fixed and dehydrated the cells in cold methanol for 5 min at −20°C. The cells were then washed three times with PBS to remove the methanol and, after the wash, they were mounted with ProLong^®^ Gold Antifade Mounting Medium with DAPI (Molecular Probes) and cover slips were added. Single plane images were acquired with a Leica DM5500 B microscope (Leica Microsystems) with a 100× oil immersion phase-contrast objective. Images were analyzed using ImageJ. The kDNA size analysis was performed on binarized 8-bit format images. Particle size was measured in arbitrary units (a.u.). We included kDNA particles of >0.01 a.u. and ≤1.0 a.u. in the analysis. The significance of the change in kDNA size was calculated with an unpaired two-tailed *t*-test.

## Supplementary Material

Supplementary information

## References

[JCS211730C1] Aphasizheva I. and Aphasizhev R. (2016). U-insertion/deletion mRNA-editing holoenzyme: definition in sight. 32, 144-156. 10.1016/j.pt.2015.10.004 PMC4737982 26572691

[JCS211730C2] Aslett M., Aurrecoechea C., Berriman M., Brestelli J., Brunk B. P., Carrington M., Depledge D. P., Fischer S., Gajria B., Gao X. et al. (2009). TriTrypDB: a functional genomic resource for the Trypanosomatidae. 38, 457-462. 10.1093/nar/gkp851 PMC2808979 19843604

[JCS211730C3] Blum B., Bakalara N. and Simpson L. (1990). A model for RNA editing in kinetoplastid mitochondria: RNA molecules transcribed from maxicircle DNA provide the edited information. 60, 189-198. 10.1016/0092-8674(90)90735-W 1688737

[JCS211730C4] Bruhn D. F., Mozeleski B., Falkin L. and Klingbeil M. M. (2010). Mitochondrial DNA polymerase POLIB is essential for minicircle DNA replication in African trypanosomes. 75, 1414-1425. 10.1111/j.1365-2958.2010.07061.x 20132449

[JCS211730C5] Bruhn D. F., Sammartino M. P. and Klingbeil M. M. (2011). Three mitochondrial DNA polymerases are essential for kinetoplast DNA replication and survival of bloodstream form Trypanosoma brucei. 10, 734-743. 10.1128/EC.05008-11 PMC3127672 21531873

[JCS211730C6] Burkard G., Fragoso C. M. and Roditi I. (2007). Highly efficient stable transformation of bloodstream forms of Trypanosoma brucei. 153, 220-223. 10.1016/j.molbiopara.2007.02.008 17408766

[JCS211730C7] Chen J., Rauch C. A., White J. H., Englund P. T. and Cozzarelli N. R. (1995). The topology of the kinetoplast DNA network. 80, 61-69. 10.1016/0092-8674(95)90451-4 7813018

[JCS211730C8] Dean S., Gould M. K., Dewar C. E. and Schnaufer A. C. (2013). Single point mutations in ATP synthase compensate for mitochondrial genome loss in trypanosomes. 110, 14741-14746. 10.1073/pnas.1305404110 PMC3767566 23959897

[JCS211730C9] Downey N., Hines J. C., Sinha K. M. and Ray D. S. (2005). Mitochondrial DNA Ligases of Trypanosoma brucei. 4, 765-774. 10.1128/EC.4.4.765-774.2005 PMC1087824 15821136

[JCS211730C10] Drew M. E. and Englund P. T. (2001). Intramitochondrial location and dynamics of Crithidia fasciculata kinetoplast minicircle replication intermediates. 153, 735-744. 10.1083/jcb.153.4.735 PMC2192374 11352935

[JCS211730C11] Engel M. L. and Ray D. S. (1999). The kinetoplast structure-specific endonuclease I is related to the 5′ exo/endonuclease domain of bacterial DNA polymerase I and colocalizes with the kinetoplast topoisomerase II and DNA polymerase beta during replication. 96, 8455-8460. 10.1073/pnas.96.15.8455 PMC17537 10411896

[JCS211730C12] Foucher A. L., Papadopoulou B. and Ouellette M. (2006). Prefractionation by digitonin extraction increases representation of the cytosolic and intracellular proteome of Leishmania infantum. 5, 1741-1750. 10.1021/pr060081j 16823982

[JCS211730C13] Gheiratmand L., Brasseur A., Zhou Q. and He C. Y. (2013). Biochemical characterization of the bi-lobe reveals a continuous structural network linking the bi-lobe to other single-copied organelles in Trypanosoma brucei. 288, 3489-3499. 10.1074/jbc.M112.417428 PMC3561568 23235159

[JCS211730C14] Göringer H. U. (2012). “Gestalt,” composition and function of the Trypanosoma brucei editosome. 66, 65-82. 10.1146/annurev-micro-092611-150150 22994488

[JCS211730C15] Gunasekera K., Wüthrich D., Braga-Lagache S., Heller M. and Ochsenreiter T. (2012). Proteome remodelling during development from blood to insect-form Trypanosoma brucei quantified by SILAC and mass spectrometry. 13, 556 10.1186/1471-2164-13-556 PMC3545838 23067041

[JCS211730C16] Hajduk S. and Ochsenreiter T. (2010). RNA editing in kinetoplastids. 7, 229-236. 10.4161/rna.7.2.11393 20220308

[JCS211730C17] Hines J. C. and Ray D. S. (2010). A mitochondrial DNA primase is essential for cell growth and kinetoplast DNA replication in Trypanosoma brucei. 30, 1319-1328. 10.1128/MCB.01231-09 PMC2832486 20065037

[JCS211730C18] Hines J. C. and Ray D. S. (2011). A second mitochondrial DNA primase is essential for cell growth and kinetoplast minicircle DNA replication in Trypanosoma brucei. 10, 445-454. 10.1128/EC.00308-10 PMC3067476 21257796

[JCS211730C19] Hoffmann A., Käser S., Jakob M., Amodeo S., Peitsch C., Týč J., Vaughan S., Zuber B., Schneider A. and Ochsenreiter T. (2018). Molecular model of the mitochondrial genome segregation machinery in *Trypanosoma brucei*. 115, E1809-E1818. 10.1073/pnas.1716582115 PMC5828607 29434039

[JCS211730C20] Hong M. and Simpson L. (2003). Genomic organization of Trypanosoma brucei kinetoplast DNA minicircles. 154, 265-279. 10.1078/143446103322166554 13677453

[JCS211730C21] Jakob M., Hoffmann A., Amodeo S., Peitsch C., Zuber B. and Ochsenreiter T. (2016). Mitochondrial growth during the cell cycle of Trypanosoma brucei bloodstream forms. 6, 36565 10.1038/srep36565 PMC5118809 27874016

[JCS211730C22] Jensen R. E. and Englund P. T. (2012). Network news: the replication of kinetoplast DNA. 66, 473-491. 10.1146/annurev-micro-092611-150057 22994497

[JCS211730C23] Kalidas S., Li Q. and Phillips M. A. (2011). A Gateway^®^ compatible vector for gene silencing in bloodstream form Trypanosoma brucei. 178, 51-55. 10.1016/j.molbiopara.2011.03.002 PMC3101333 21420443

[JCS211730C24] Käser S., Oeljeklaus S., Týč J., Vaughan S., Warscheid B. and Schneider A. (2016). Outer membrane protein functions as integrator of protein import and DNA inheritance in mitochondria. 113, E4467-E4475. 10.1073/pnas.1605497113 PMC4978248 27436903

[JCS211730C25] Käser S., Willemin M., Schnarwiler F., Schimanski B., Poveda-Huertes D., Oeljeklaus S., Warscheid B., Meisinger C. and Schneider A. (2017). Biogenesis of a mitochondrial DNA inheritance machinery in the mitochondrial outer membrane. 13, 1-26. 10.1371/journal.ppat.1006808 PMC5764417 29287109

[JCS211730C26] Kilmartin J. V., Wright B. and Milstein C. (1982). Rat monoclonal antitubulin antibodies derived by using a new nonsecreting rat cell line. 93, 576-582. 10.1083/jcb.93.3.576 PMC2112140 6811596

[JCS211730C27] Klingbeil M. M. and Englund P. T. (2004). Closing the gaps in kinetoplast DNA network replication. 101, 4333-4334. 10.1073/pnas.0401400101 PMC384744 15070715

[JCS211730C28] Klingbeil M. M., Motyka S. A. and Englund P. T. (2002). Multiple mitochondrial DNA polymerases in Trypanosoma brucei. 10, 175-186. 10.1016/S1097-2765(02)00571-3 12150917

[JCS211730C29] Laurent M. and Steinert M. (1970). Electron microscopy of kinetoplastic DNA from Trypanosoma mega. 66, 419-424. 10.1073/pnas.66.2.419 PMC283061 5271173

[JCS211730C30] Li Y., Sun Y., Hines J. C. and Ray D. S. (2007). Identification of new kinetoplast DNA replication proteins in Trypanosomatids based on predicted S-phase expression and mitochondrial targeting. 6, 2303-2310. 10.1128/EC.00284-07 PMC2168261 17965251

[JCS211730C31] Liu B., Molina H., Kalume D., Pandey A., Griffith J. D. and Englund P. T. (2006). Role of p38 in replication of Trypanosoma brucei kinetoplast DNA. 26, 5382-5393. 10.1128/MCB.00369-06 PMC1592711 16809774

[JCS211730C32] Liu B., Yildirir G., Wang J., Tolun G., Griffith J. D. and Englund P. T. (2010). TbPIF1, a Trypanosoma brucei mitochondrial DNA helicase, is essential for kinetoplast minicircle replication. 285, 7056-7066. 10.1074/jbc.M109.084038 PMC2844155 20042610

[JCS211730C33] Lukeš J., Hines J. C., Evans C. J., Avliyakulov N. K., Prabhu V. P., Chen J. and Ray D. S. (2001). Disruption of the Crithidia fasciculata KAP1 gene results in structural rearrangement of the kinetoplast disc. 117, 179-186. 10.1016/S0166-6851(01)00348-6 11606228

[JCS211730C34] McDermott S. M., Luo J., Carnes J., Ranish J. A. and Stuart K. (2016). The Architecture of Trypanosoma brucei editosomes. 113, E6476-E6485. 10.1073/pnas.1610177113 PMC5081628 27708162

[JCS211730C35] Milman N., Motyka S. A., Englund P. T., Robinson D. and Shlomai J. (2007). Mitochondrial origin-binding protein UMSBP mediates DNA replication and segregation in trypanosomes. 104, 19250-19255. 10.1073/pnas.0706858104 PMC2148276 18048338

[JCS211730C36] Oberholzer M., Morand S., Kunz S. and Seebeck T. (2006). A vector series for rapid PCR-mediated C-terminal in situ tagging of Trypanosoma brucei genes. 145, 117-120. 10.1016/j.molbiopara.2005.09.002 16269191

[JCS211730C37] Ochsenreiter T., Cipriano M. and Hajduk S. L. (2007). KISS: the kinetoplastid RNA editing sequence search tool. 13, 1-4. 10.1261/rna.232907 PMC1705751 17123956

[JCS211730C38] Ogbadoyi E. O. (2003). A high-order trans-membrane structural linkage is responsible for mitochondrial genome positioning and segregation by flagellar basal bodies in trypanosomes. 14, 1769-1779. 10.1091/mbc.E02-08-0525 PMC165075 12802053

[JCS211730C39] Peikert C. D., Mani J., Morgenstern M., Käser S., Knapp B., Wenger C., Harsman A., Oeljeklaus S., Schneider A. and Warscheid B. (2017). Charting organellar importomes by quantitative mass spectrometry. 8, 15272 10.1038/ncomms15272 PMC5436138 28485388

[JCS211730C40] Povelones M. L. (2014). Beyond replication: division and segregation of mitochondrial DNA in kinetoplastids. 196, 53-60. 10.1016/j.molbiopara.2014.03.008 24704441

[JCS211730C41] Robertson M. (1913). Notes on the life-history of Trypanosoma gambiense, with a brief reference to the cycles of Trypanosoma nanum and trypanosoma pecorum in glossina palpalis. B203, 161-184. 10.1098/rstb.1913.0005

[JCS211730C42] Robinson D. R. and Gull K. (1991). Basal body movements as a mechanism for mitochondrial genome segregation in the trypanosome cell cycle. 352, 731-733. 10.1038/352731a0 1876188

[JCS211730C43] Saxowsky T. T., Choudhary G., Klingbeil M. M. and Englund P. T. (2003). Trypanosoma brucei has two distinct mitochondrial DNA polymerase beta enzymes. 278, 49095-49101. 10.1074/jbc.M308565200 12966090

[JCS211730C44] Schimanski B., Nguyen T. N., Günzl A. and Gu A. (2005). Highly efficient tandem affinity purification of trypanosome protein complexes based on a novel epitope combination highly efficient tandem affinity purification of trypanosome protein complexes based on a novel epitope combination. 4, 1942-1950. 10.1128/EC.4.11.1942-1950.2005 PMC1287860 16278461

[JCS211730C45] Schnarwiler F., Niemann M., Doiron N., Harsman A., Kaser S., Mani J., Chanfon A., Dewar C. E., Oeljeklaus S., Jackson C. B. et al. (2014). Trypanosomal TAC40 constitutes a novel subclass of mitochondrial -barrel proteins specialized in mitochondrial genome inheritance. 111, 7624-7629. 10.1073/pnas.1404854111 PMC4040615 24821793

[JCS211730C46] Simpson L. (1968). Behavior of the kinetoplast of leishmania tarentolae upon cell rupture. 15, 132-136. 10.1111/j.1550-7408.1968.tb02097.x 4868432

[JCS211730C47] Trikin R., Doiron N., Hoffmann A., Haenni B., Jakob M., Schnaufer A., Schimanski B., Zuber B. and Ochsenreiter T. (2016). TAC102 is a novel component of the mitochondrial genome segregation machinery in trypanosomes. 12, e1005586 10.1371/journal.ppat.1005586 PMC4864229 27168148

[JCS211730C48] Urbaniak M. D., Martin D. M. A. and Ferguson M. A. J. (2013). Global quantitative SILAC phosphoproteomics reveals differential phosphorylation is widespread between the procyclic and bloodstream form lifecycle stages of Trypanosoma brucei. 12, 2233-2244. 10.1021/pr400086y PMC3646404 23485197

[JCS211730C49] Wang Z. and Englund P. T. (2001). RNA interference of a trypanosome topoisomerase II causes progressive loss of mitochondrial DNA. 20, 4674-4683. 10.1093/emboj/20.17.4674 PMC125608 11532932

[JCS211730C50] Wang Z., Morris J. C., Drew M. E. and Englund P. T. (2000). Inhibition of Trypanosoma brucei gene expression by RNA interference using an integratable vector with opposing T7 promoters. 275, 40174-40179. 10.1074/jbc.M008405200 11013266

[JCS211730C51] Wirtz E., Leal S., Ochatt C. and Cross G. A. M. (1999). A tightly regulated inducible expression system for conditional gene knock-outs and dominant-negative genetics in *Trypanosoma brucei*. 99, 89-101. 10.1016/S0166-6851(99)00002-X 10215027

[JCS211730C52] Woods A., Sherwin T., Sasse R., MacRae T. H., Baines A. J. and Gull K. (1989). Definition of individual components within the cytoskeleton of Trypanosoma brucei by a library of monoclonal antibodies. 93, 491-500. 10.1242/jcs.93.3.491 2606940

[JCS211730C53] Xu C. W., Hines J. C., Engel M. L., Russell D. G. and Ray D. S. (1996). Nucleus-encoded histone H1-like proteins are associated with kinetoplast DNA in the trypanosomatid Crithidia fasciculata. 16, 564-576. 10.1128/MCB.16.2.564 PMC231035 8552084

[JCS211730C54] Zhang X., Cui J., Nilsson D., Gunasekera K., Chanfon A., Song X., Wang H., Xu Y. and Ochsenreiter T. (2010). The Trypanosoma brucei MitoCarta and its regulation and splicing pattern during development. 38, 7378-7387. 10.1093/nar/gkq618 PMC2995047 20660476

[JCS211730C55] Zhao Z., Lindsay M. E., Roy Chowdhury A., Robinson D. R. and Englund P. T. (2008). p166, a link between the trypanosome mitochondrial DNA and flagellum, mediates genome segregation. 27, 143-154. 10.1038/sj.emboj.7601956 PMC2206137 18059470

