# Supplementary material for: Characterization of the novel mitochondrial genome replication factor MiRF172 in Trypanosoma brucei
Source: J Cell Sci. 2018 Apr 25;131(8):jcs211730. doi: 10.1242/jcs.211730 (PMC5963845; doi:10.1242/jcs.211730)
Supplement: Supplementary information [file joces-131-211730-s1.pdf]

Supplementary Material

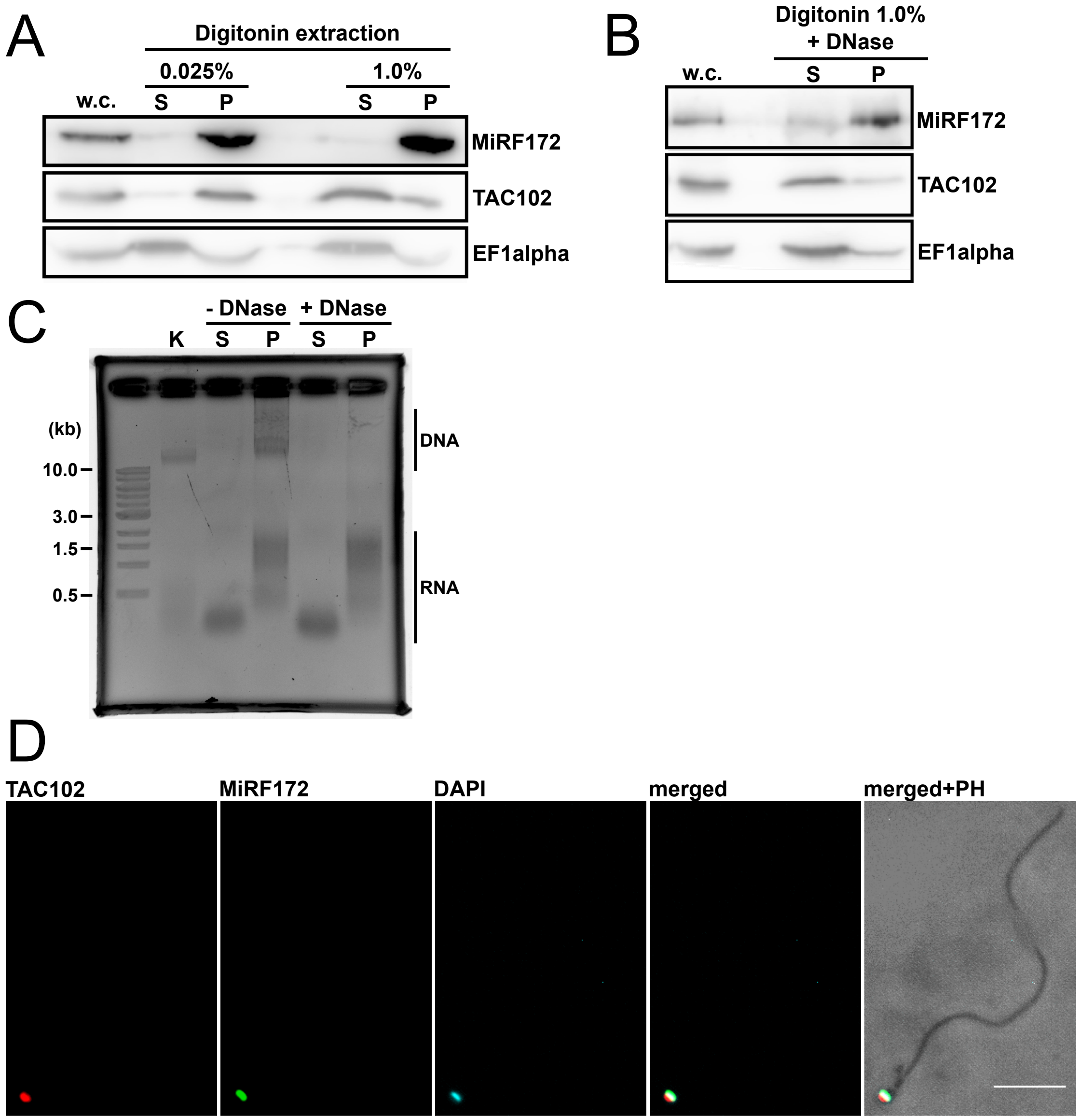

Fig. S1 | Biochemical characterization of MiRF172 by detergent and high salt extractions. A) Western blot of digitonin fractions with different digitonin concentrations and without DNaseI treatment of BSF cells expressing MiRF172-PTP. MiRF172-PTP was detected with PAP antibody. TAC102, a mitochondrial protein, and EF1alpha, a cytosolic protein, were used as controls of the fractionation. **B)** Western blot from digitonin fractionations with 1% digitonin and DNaseI treatment. Detection of proteins was performed with the same antibodies as in B). **C)** Agarose gel of DNA from 1% digitonin fractionation either without or with DNaseI treatment. **D)** Localization of MiRF172-PTP in flagellar extracts. MiRF172-PTP (green) was detected by the anti-Protein A antibody. TAC102 was detected with the anti-TAC102 monoclonal mouse antibody (red). The kDNA was stained with DAPI (cyan). w.c. = whole cells, S = supernatant, P = pellet, K = NYsm control DNA. Scale bar = 5 µm.

Journal of Cell Science • Supplementary information

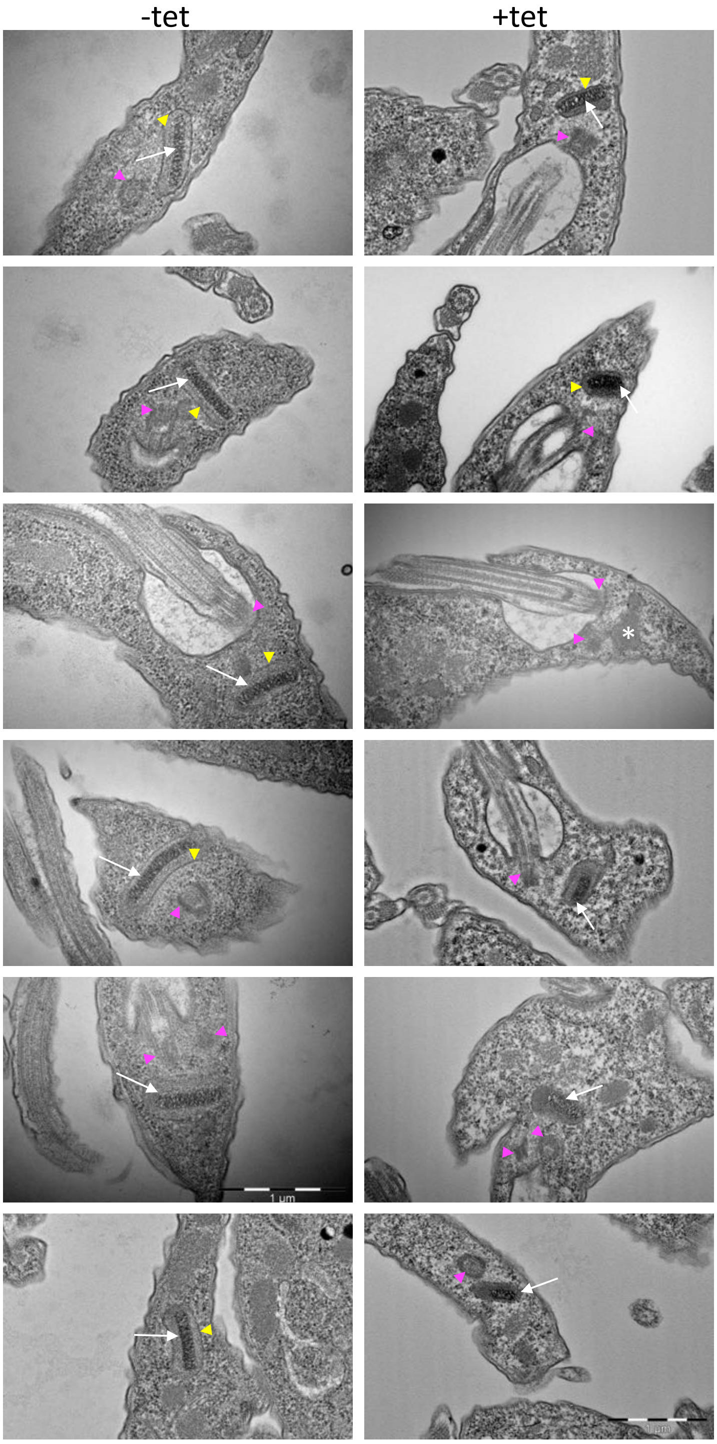

Fig. S2 l Representative thin section TEM imagery of kDNA from MiRF172 RNAi in BSF cells. On the left side kDNAs from uninduced cells (-tet) are shown. Representative images of kDNAs from cells at day three post induction (+tet) are shown on the right side. The white asterisk marks a kDNA pocket without kDNA (0K cell). The white arrows point to the kDNA, yellow arrowheads to the mitochondrial membrane and the magenta arrowheads to basal body or base of flagellum. Scale bar = 1 µm.

Journal of Cell Science • Supplementary information

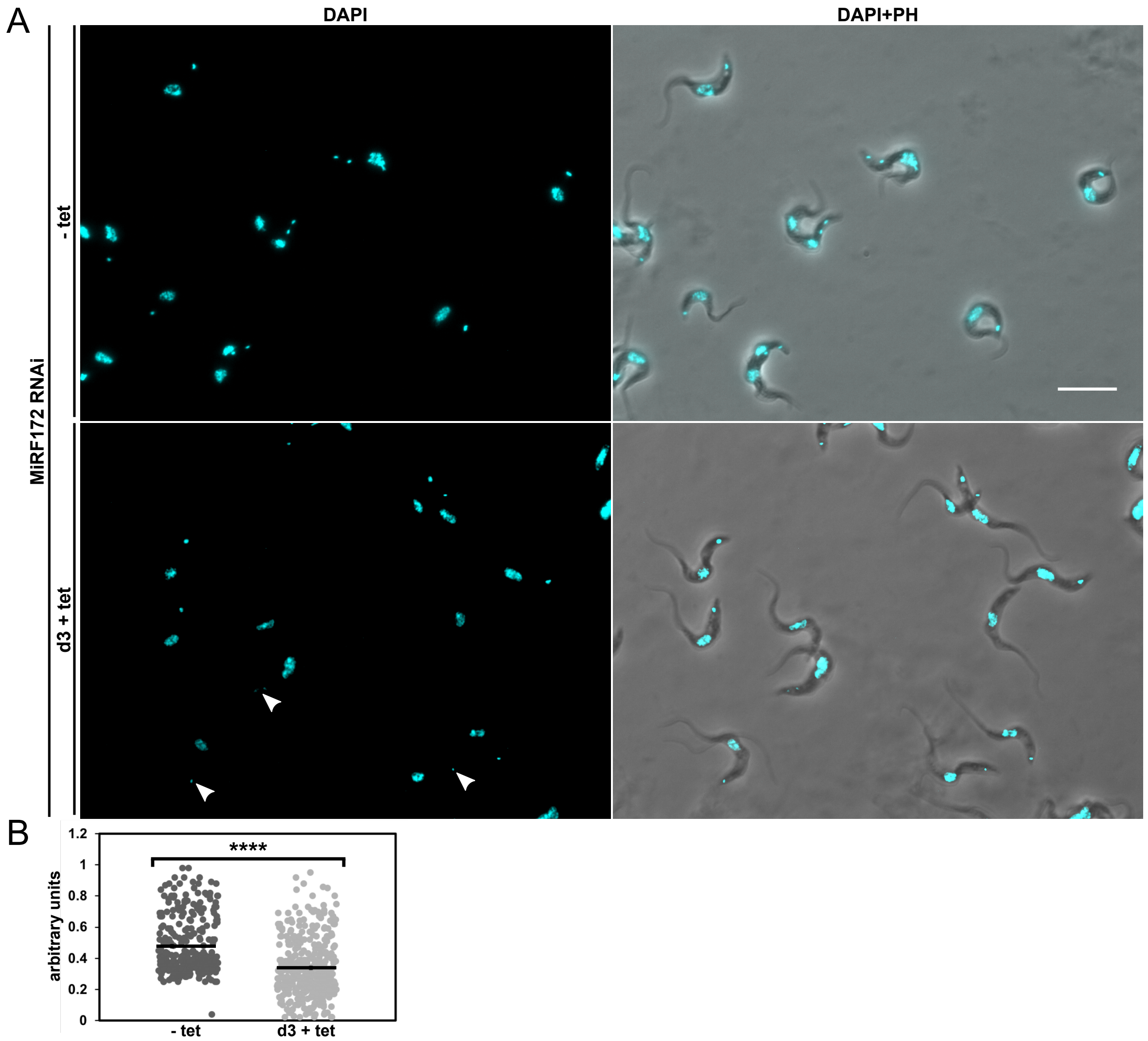

Fig. S3 I Small kDNA phenotype upon knockdown of MiRF172 mRNA by RNAi in BSF *T. brucei* cells. A) Representative images of MiRF172 RNAi cells either without uninduced (- tet) or at day three of RNAi induction (d3 + tet). The kDNA and the nucleus were stained with DAPI (cyan). PH = phase contrast, scale bar = 10 µm. **B)** Size measurements (n ≥ 297 for each condition) of kDNA DAPI signals from uninduced (- tet; mean = 0.476 arbitrary units) and induced (day3 + tet; mean = 0.337 arbitrary units) MiRF172 RNAi BSF cells. Significance of difference in size was calculated by two-tailed unpaired t- test. **** = p ≤ 0.0001

Journal of Cell Science • Supplementary information

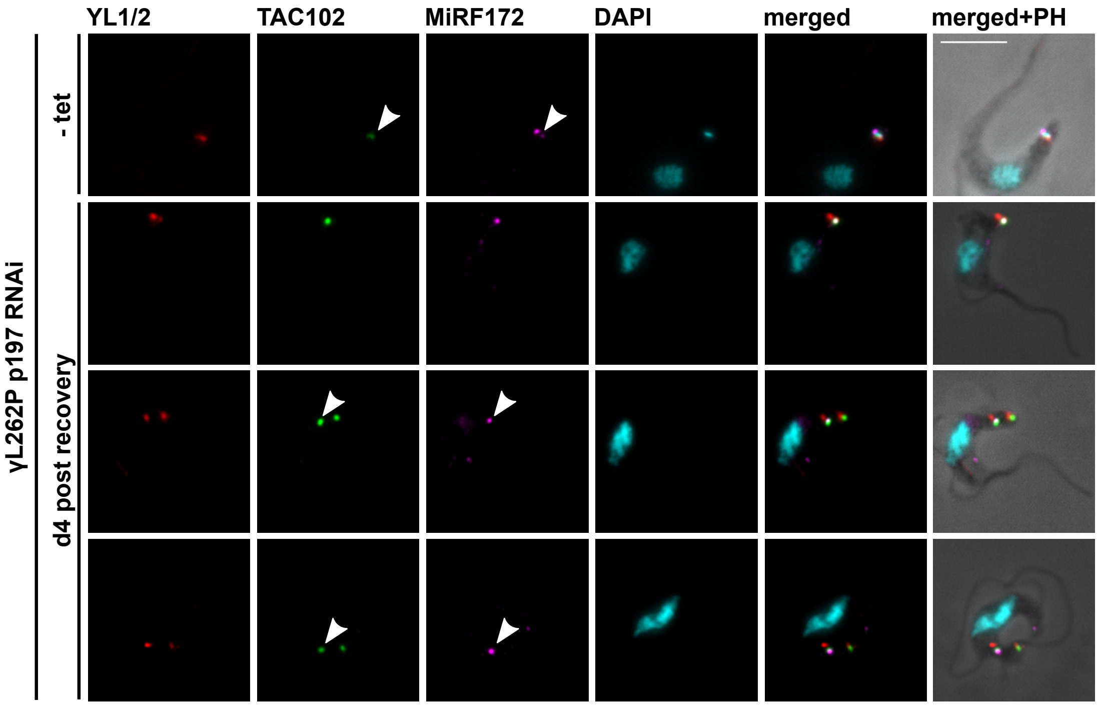

Fig. S4 | Representative immunofluorescence microscopy images showing MiRF172 and TAC102 after recovery from p197 RNAi in γL262P BSF *T. brucei* cells. Localization of MiRF172-PTP (magenta), TAC102 (green) represented by maximum intensity projections from immunofluorescence microscopy image stacks of γL262P p197 RNAi BSF *T. brucei* cells either uninduced (- tet) or at day four after removal of tet (d4 post recovery). MiRF172-PTP was detected by the anti-Protein A antibody. TAC102 was detected with the anti-TAC102 monoclonal mouse antibody (green). The kDNA and the nucleus were stained with DAPI (cyan). PH = phase contrast, scale bar = 5 µm.

Journal of Cell Science • Supplementary information
